# Effects of acute hypoxia on heart rate variability, sample entropy and cardiorespiratory phase synchronization

**DOI:** 10.1186/1475-925X-13-73

**Published:** 2014-06-11

**Authors:** Da Zhang, Jin She, Zhengbo Zhang, Mengsun Yu

**Affiliations:** 1 School of Biological Science and Medical Engineering, Beihang University, Beijing, China; 2 Department of Biomedical Engineering, Chinese PLA (People’s Liberation Army) General Hospital, Beijing, China; 3 Research Center of Aviation Medicine Engineering, Institute of Aviation Medicine, Beijing, China

**Keywords:** Hypoxia, Autonomic nervous system, Heart rate variability, Sample entropy, Cardiorespiratory phase synchronization

## Abstract

**Background:**

Investigating the responses of autonomic nervous system (ANS) in hypoxia may provide some knowledge about the mechanism of neural control and rhythmic adjustment. The integrated cardiac and respiratory system display complicated dynamics that are affected by intrinsic feedback mechanisms controlling their interaction. To probe how the cardiac and respiratory system adjust their rhythms in different simulated altitudes, we studied heart rate variability (HRV) in frequency domain, the complexity of heartbeat series and cardiorespiratory phase synchronization (CRPS) between heartbeat intervals and respiratory cycles.

**Methods:**

In this study, twelve male subjects were exposed to simulated altitude of sea level, 3000 m and 4000 m in a hypobaric chamber. HRV was assessed by power spectral analysis. The complexity of heartbeat series was quantified by sample entropy (SampEn). CRPS was determined by cardiorespiratory synchrogram.

**Results:**

The power spectral HRV indices at all frequency bands depressed according to the increase of altitude. The SampEn of heartbeat series increased significantly with the altitude (P < 0.01). The duration of CRPS epochs at 3000 m was not significantly different from that at sea level. However, it was significantly longer at 4000 m (P < 0.01).

**Conclusions:**

Our results suggest the phenomenon of CRPS exists in normal subjects when they expose to acute hypoxia. Further, the autonomic regulation has a significantly stronger influence on CRPS in acute hypoxia. The changes of CRPS and HRV parameters revealed the different regulatory mechanisms of the cardiac and respiratory system at high altitude.

## Background

Nowadays, advanced transport technology gives people more opportunity to visit high altitude, such as Tibet. However, most visitors who have not enough time to acclimatize to the hypoxic environment may have some risk for physical problems, including cardiovascular disorders [1]. Burtscher [2] demonstrated that up to 30% of all deaths in mountain sports at altitude were ascribed to sudden cardiac death. Hypoxia induces tachycardia when oxygen concentration is lower than 17% [3]. In addition, moderate altitude could increase the incidence of cardiac arrhythmia in healthy older people [4]. Kujanik et al. [5] also reported the occurrence of supraventricular and ventricular extrasystoles was proportional to the altitude in acute hypoxia in healthy older man. These findings suggest that hypoxia-induced changes in cardiac rhythm may be a threat for the health of people exposed to hypoxic environment.

The responses of autonomic nervous system (ANS) are crucial for acclimatization to hypoxia. Acute hypoxia activates several autonomic mechanisms, mainly in cardiovascular system such as increasing in resting heart rate (HR), cardiac output and blood pressure [6,7], and in respiratory system like causing pulmonary hypertension and hyperventilation [8]. Hypoxic exposure is a potent activator of ANS [9]. The responses of ANS are usually evaluated by heart rate variability (HRV). Many researchers employed power spectral technique to estimate power distribution as a function of frequency. In this method, the power spectral density of R-R interval (RRI) series is used to quantified to three main spectral power components: very low frequency (VLF, 0–0.04 Hz), low frequency (LF, 0.04-0.15 Hz) and high frequency (HF, 0.15-0.4 Hz) [10]. HF power components are associated with cardiac parasympathetic activity, whereas LF power components reflect both sympathetic and parasympathetic activities [10]. The LF/HF ratio is an index of sympathovagal balance or the reflection of sympathetic modulations [10]. VLF components do not have explicit physiological properties and should be avoided in short term HRV analysis [10].

Generally, the linear methods on HRV analysis including time- and frequency-domain have been widely used in hypoxia, because the results of linear approaches are easy to interpret in physiologic terms. But they also have some limitations, and their results are inconsistent. Most studies indicated that both LF and HF power decreased at high altitude [1,6,9,11-16]. However, some other studies showed that the increase of LF power was accompanied with the decrease of HF power [17], or unchanged HF power was concomitant with the increase of LF power [3]. These discrepancies may attribute to the complicated fluctuations of the sinus rhythm and multiple feedback control in cardiovascular regulation. Meanwhile, it has been demonstrated that some nonlinear processes are involved in the regulation of the cardiovascular and respiratory system, especially in extreme conditions [18]. Therefore, we could lose a lot of information about cardiac complex dynamics when we analyze heartbeat series with traditional linear methods. On the other hand, nonlinear changes of heart rate time series are determined by the complicated interactions of haemodynamic, electrophysiological and humoral variables [10]. It has been speculated that the analysis of heartbeat series based on nonlinear approaches may provide complementary and extra information about how cardiovascular system regulates. Therefore, it is necessary to combine linear and nonlinear approaches to analyze heartbeat series in an attempt to characterize cardiovascular regulation during hypoxic exposure. Sample entropy (SampEn) has been used to examine complexity or irregularity of heartbeat series. Nonetheless, the interpretation of nonlinear properties such as SampEn is not completely clear because there are only few studies referring to the nonlinear parameters of cardiovascular changes in different physiological states or environmental stimuli.

Compared with the univariate analysis, bivariate method may provide more detail information about the neural regulatory mechanism. Cardiovascular and respiratory system are functionally integrated by neural regulation and intrinsic feedback mechanisms. It is necessary to determine interactions between the two key systems. The interactions between cardiac and respiratory system are traditionally identified by respiratory sinus arrhythmia (RSA), which represents HR acceleration during inspiration and deceleration during expiration [10]. Recently, nonlinear dynamics and mathematical physics have been developed to quantify cardiorespiratory coupling through phase synchronization between cardiac series and respiratory signal [19-21]. Earlier studies had quantitatively assessed the changes of cardiorespiratory phase synchronization (CRPS) in different states, like vigorous in Zen meditation [22] and in reciting hexameter verse [23], diminished during strain [24] and mental task [25]. Moreover, CRPS is dramatically increased in non-rapid eye movement sleep [26] and reduced in elder subjects [27]. Both cardiac and respiratory dynamics display long-term transient changes related to different physiological states and environmental stimuli. How CRPS responds to acute hypoxia in association with underlying mechanisms of physiological control remains unclear.

In the present study, to evaluate the changes of ANS in acute hypoxia, we investigated how acute exposure to simulated altitude of 3000 m and 4000 m influenced HRV in frequency domain. On the other hand, exploring SampEn of heartbeat series in acute hypoxia could help us to understand the autonomic regulation of cardiac dynamics. We hypothesized that cardiorespiratory coupling might undergo phase transitions with the changes of physiological stress. At the same time, we are curious about their phase transitions in different simulated altitudes. We investigated the variations of cardiorespiratory coupling through phase synchronization during transitions from one physiologic state (normoxia) to another (hypoxia).

## Methods

### Subjects and experimental protocol

Twelve rigorously screened healthy subjects participated in this study. The mean age, height and body mass was 29 ± 7 year, 172.54 ± 4.97 cm and 71.08 ± 9.11 kg (mean ± SD), respectively. None of them had ever been to high altitude site above 2000 m within six months before the experiment. All the subjects were required to avoid drinking alcohol or beverage with caffeine within 12 hours before this experiment. The protocol of this study was approved by the Ethics Committee of Beihang University and all subjects gave informed consent to take part in the study.

**Figure 1:**
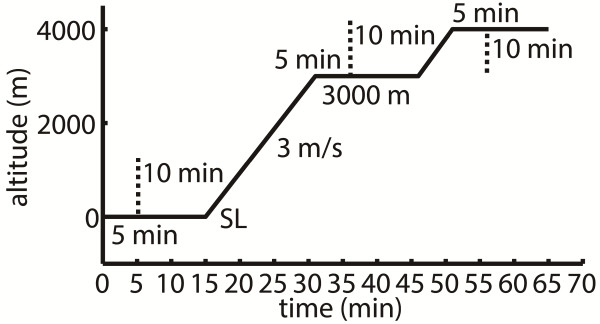
**Experimental protocol described with a diagram showing altitude vs. time.** Each subject stayed at each simulated altitude for 15 minutes. The physiological data of the last 10 minutes was considered as steady state and adopted for analysis. The simulated altitude ascended from sea level (SL) to 3000 m and 3000 m to 4000 m at the rate of 3 m/s.

This study was conducted in a hypobaric hypoxic chamber (Institute of Aviation Medicine, Beijing, China) with a volume of more than 20 m^3^ (length, width, and height is 6 m, 2 m, and 1.8 m, respectively). The chamber is situated at 31.3 m height as the sea level (SL), which is the altitude of Beijing, with the atmospheric pressure approximating 101 kPa (China Meteorological Data Sharing Service System, http://cdc.cma.gov.cn). The chamber is able to lower atmospheric pressure to simulate the altitude of 5500 m (approximately 51 kPa).In this study, three levels of altitude were simulated: SL, 3000 m (approximately 70 kPa) and 4000 m (approximately 62kPa). The simulated altitude changed between each other at a rate of 3 m/s. Each subject stayed at each simulated altitude for 15 minutes (Figure 1). The physiological variables of the last 10 minutes was considered as steady state and adopted for analysis. During the experiment, all subjects were required to stay in the chamber quietly and breathe spontaneously in a seating position. Throughout the whole experiment, temperature in the chamber was kept constant at 22°C.

Physiological signals of ECG, respiration and arterial oxygen saturation (SpO2) were monitored during the entire experiment. ECG was acquired by standard Ag/AgCl electrodes from right flank to left (modified 3-lead) and sampled by a 16-bit A/D converter at 1000 Hz. Respiration was recorded by electrical impedance pneumograph from ECG electrodes simultaneously. SpO2 was monitored by a finger pulse oximeter (Radical-7, Masimo, CA, USA).

### Data analysis

All data were analyzed off-line in MATLAB (The MathWorks Inc., Natick, MA, USA). At each altitude, the mean value of HR, respiratory rate (RespR) and SpO2 was calculated. At the same time, linear and nonlinear indices, reflecting the regulatory mechanism between cardiovascular and respiratory systems, such as HRV and CRPS, were investigated.

For accurate QRS complex detection, the raw ECG waveforms were filtered by a linear phase finite impulse response filter with pass-band 10–25 Hz [28] to remove power line interference, high frequency noise and baseline wander. ECG beat detection was performed using Hamilton & Tompins’ QRS detector [29] and each beat annotation was visually inspected. Then, R-waves were identified from QRS complexes. RRI time series was obtained from consecutive R peaks. HR was calculated based on R-R intervals.

The raw respiratory signal was filtered by a linear phase finite impulse response filter with the pass-band 0.1-1.0 Hz to assure the signal was a narrow-band signal. For respiratory rate (RespR) detection, the troughs and peaks of the respiratory curve were used as indicators of the onsets of inspiration and expiration, respectively.

### Spectral HRV analysis

HRV was assessed by both linear (power spectral analysis) and nonlinear (sample entropy) method. The RRI time series was firstly interpolated to 4 Hz to provide equidistant data points. The resulting RRI series was band-pass filtered to remove components below 0.015 Hz and fluctuations above the Nyquist frequency (2 Hz). The power spectral density of RRI was estimated by the Welch’s periodogram method. We applied a Hamming window of 1024 points length for each data segments, shifted by 512 points overlap. The spectral power was evaluated for each subject as the integrated area under the power spectrum curve in LF (0.04-0.15 Hz) and HF (0.15-0.4 Hz) ranges. The ratio of LF power to HF power (LF/HF) was also calculated.

### Sample entropy analysis

Besides the power spectral analysis, RRI time series was also analyzed by nonlinear dynamics method. After removing the linear trend, SampEn was introduced to quantify the complexity of RRI series at different simulated altitudes.

SampEn is defined as the negative natural logarithm of the conditional probability that two sequences similar for *l* points remain similar at the next point within a tolerance *r*, where self-matches are not included in calculating the probability [30]. An irregular sequence will conduce to larger SampEn values, whereas regular signal is associated with lower SampEn. The expression of SampEn is $\mathrm{SampEn}\left(r,l\right)=-ln\left(\frac{A}{B}\right)$, where *A* and *B* are the total numbers of forward matches of length *l* + 1 and *l*[30]. In theory, SampEn does not depend on the length of time series. Although *l* and *r* critically affect the result of SampEn, there are no guidelines for optimal selection of their values [31]. Therefore, according to the advice of Lake et al. [31], we used the two values *l* = 2 and *r* = 0.25 × *SD*(*RRI*), where SD is the standard deviation of the 10 minutes RRI time series.

### CRPS and synchrogram

In this study, we investigated the CRPS by cardiorespiratory synchrogram in all subjects at each simulated altitude. Cardiorespiratory synchrogram or synchrogram is a visual tool for inspecting synchronization between R-waves and respiratory phase. It displays the phase of respiratory signal at the times of R-peaks. The key feature of synchrogram is that the phase of a consecutive signal (respiration) is plotted at occurrences *t*_*R*_ of a second signal (R peaks in ECG at *t*_*R*_) described by a point process [27]. Parallel horizontal lines appear in phase synchrogram when cardiorespiratory phase synchronization exists.

**Figure 2:**
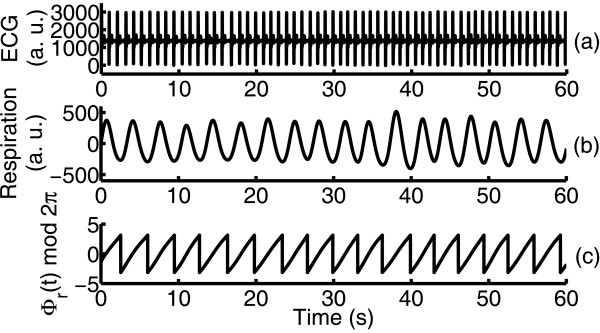
ECG (a), respiratory signal (b) and the instantaneous phase of the respiratory signal (c) for subject 5 at sea level.

**Figure 3:**
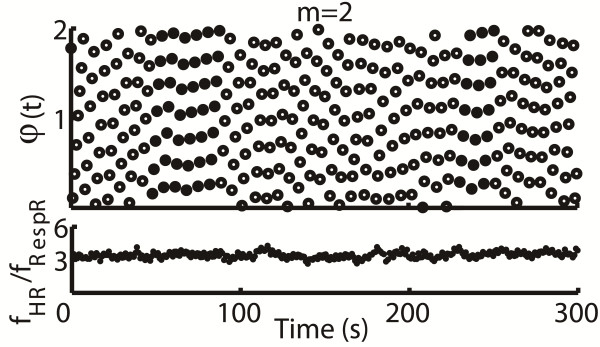
**The cardiorespiratory synchrogram for subject 5 at sea level was plotted at the top.** The solid dots located at 48 s to 88 s and 230 s to 251 s respectively composed 7 parallel lines in synchrogram and demonstrated CRPS with the ratio of 7:2 (*n* = 7 heartbeats within *m* = 2 consecutive respiratory cycles) during the 300 s periods. *f*_*HR*_/*f*_*RespR*_ which was the instantaneous ratio of heart rate (*f*_*HR*_) to respiratory rate (*f*_*RespR*_) was plotted at the bottom.

We observed the phase of respiratory signal *φ*_*b*_ at the times of the *R*^th^ R-peak *t*_*R*_, and plotted this phase versus *t*_*R*_. The instantaneous phase of respiratory signal is calculated by analytic signal approach [27]. In this way, the instantaneous respiratory phase *φ*_*b*_ represents the angle between the breathing signal and its Hilbert transform [32], which is the imaginary part of the breathing signal. The plot of *φ*_*b*_(*t*_*R*_) versus *t*_*R*_ is defined the synchrogram. In the simplest case of *n*:1 synchronization, where *n* is the number of heartbeats, there are *n* distinct values in each respiratory phase, thus, the plot would display *n* parallel horizontal lines when phase synchronization exists. In *n*:*m* locking, where *n* heartbeats occur in *m* respiratory cycles, the times *t*_*R*_ of the occurrence of R-peaks are plotted on the cumulative respiratory phase Φ_*m*_, and the respiratory phase of the *m* breathing cycles is expressed as:

$$\phi_{b}^{m}\left(t_{r}\right)=\frac{1}{2\pi }\left(\Phi_{m}\left(t_{r}\right)\mathrm{mod}2\mathrm{\pi m}\right)$$

where *t*_*R*_ is the time of the *R*^th^ heart beat and Φ_*m*_ is the cumulative respiratory phase. $\phi_{b}^{m}$ is wrapped into [0, 2*πm*] interval (Figure 2). Plotting these phase points $\phi_{b}^{m}\left(t_{R}\right)$ as a function of *t*_*R*_ would shows *n* horizontal plateaus when synchronization is present between the two systems (Figure 3). An important feature of this method is that, only one integer *m* should be selected by trial. Moreover, several synchronous regimes could be distinguished visually within one plot, and the transitions between them can be traced.

In this study, phase recurrence was used to quantify the cardiorespiratory synchrogram. This method is based on the heuristic approach [33]. In parallel horizontal lines, the relative distance of each *n*^th^ R-peak has to be approximately identical. Otherwise, the horizontal strips would not occur. A *n*:*m* phase synchronization will be detected if the discrepancy between the respiratory phase corresponding to the (*i* + *n*)^th^ R-peak and the phase corresponding to the *i*^th^ R-peak is within a predefined tolerance *ϵ*. This condition has to be conducted for at least *k* successive R-peaks.

$$\exists k>1,\left|\phi_{b}\left(t_{i+n}\right)-\phi_{b}\left(t_{i}\right)\right|<\epsilon ,i\in \left\{j,\cdots ,j+k-1|0\leq j\leq N_{r}-k+1\right\}$$

where *N*_*r*_ is the total number of R-peaks. To be compatible with the description of ‘parallel horizontal lines’ during coupling, *k* ≥ *m* needs to be fulfilled [33]. This process needs to detect the structure of parallel horizontal strips with a length of 2*n* successive normalized relative phases. For example, a 4:1 synchronization may be retrieved from at least successive 8 R-peaks. This method needs to be applied to each ratio of *n*:*m*. In our paper, phase recurrence was applied to adjacent respiratory cycles from *m* = 1 to 4. The tolerance *ϵ* was set to *ϵ* = 0.025 [33]. If one segment of synchronization was identified, the time duration of this segment was calculated. To exclude spurious detection of cardiorespiratory synchronization, only the segments of phase synchronization with time intervals more than 20s was considered. We summed the total time of the identified synchronization segments, and denoted it as the *synchronization time* (*T*).

### Statistical analysis

The results were presented as mean ± SD. Bartlett’s test was used for equal variance test. Logarithmic transform was performed on SpO2 and *T* to make data normal distribution before statistical analysis. One-way repeated ANOVA was used to compare the data at different simulated altitudes. Further difference was tested by pairwise multiple comparison with Bonferroni modification. All statistical analysis was performed in MATLAB and P value <0.05 was considered as statistical significance.

## Results

### Physiologic parameters

The mean values of SpO2, HR and RespR at each altitude were listed in Table 1. Hypoxia led to increasing resting HR and RespR accompanied with decreasing SpO2 (Table 1). Both resting HR and SpO2 were significantly changed at 4000 m compared with the value at SL and 3000 m. RespR at 3000 m was not significantly different from that at SL. However, it was significantly increased at 4000 m.

**Table 1:** SpO2, HR and RespR recorded at SL, 3000 m and 4000 m

	**SL**	**3000 m**	**4000 m**
SpO2 (%)	97 ± 1	90 ± 3	84 ± 4 §
HR (1/min)	72 ± 5	77 ± 5	84 ± 5 ‡
RespR (1/min)	22 ± 2	23 ± 2	24 ± 2 †

§ Significantly lower compared with the value at SL and 3000 m (both P < 0.001).

‡ Significantly increase compared with the value at SL (P < 0.001) and 3000 m (P = 0.002).

† Significantly change compared to the value at SL (P = 0.012), but there was no significant change between SL and 3000 m (P = 0.169), neither between 3000 m and 4000 m (P = 0.217).

### HRV parameters

The results of HRV analysis at different altitudes were shown in Table 2. Both LF and HF power decreased dramatically with the increase of altitude. Significant increase in the LF/HF ratio suggested HF power was suppressed much more than LF power. This result indicated the activities of ANS were attenuated in acute hypoxia and sympathovagal balance shifted to sympathetic dominance. Nonlinear analysis displayed a significant increase in SampEn according to the ascent of altitude, revealing a higher irregularity of cardiac rhythm in acute hypoxia.

**Table 2:** HRV indices recorded at different altitudes

	**SL**	**3000 m**	**4000 m**
LF(ms^2^)	623 ± 290	427 ± 192	253 ± 137 ¶
HF(ms^2^)	754 ± 649	473 ± 517	177 ± 266 §
LF/HF	1.2 ± 0.8	1.9 ± 1.7	2.7 ± 1.3 ‡
SampEn	1.7 ± 0.1	1.8 ± 0.1 †	1.9 ± 0.1 †

¶ Significantly decreased compared with the value at SL (P < 0.001) and 3000 m (P = 0.008).

§ Significantly reduced compared to the value at SL (P < 0.001) and 3000 m (P = 0.007).

‡ Significantly increased from SL to 4000 m (P = 0.006), but there was no significant difference between SL and 3000 m (P = 0.062), neither between 3000 m and 4000 m (P = 0.321).

† Significantly increased compared with the value at SL (P = 0.004 at 3000 m and P < 0.001 at 4000 m). However, there was no significant difference between 3000 m and 4000 m (P = 0.127).

### CRPS

**Figure 4:**
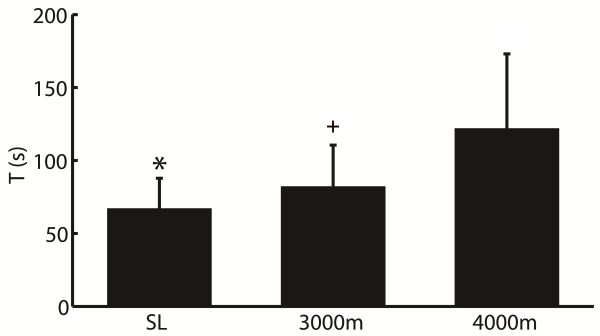
**Synchronization time *T *(s) changed with the simulated altitude.** The value was 60 ± 26 s, 80 ± 41 s and 113 ± 48 s at sea level (SL), 3000 m and 4000 m, respectively. The *T* at 4000 m was significantly longer than the value at SL (asterisk indicates P = 0.003) and 3000 m (plus indicates P = 0.040), but there was non-significantly change between at 3000 m and at SL (P = 0.214). The error bars indicated the standard deviation.

For cardiorespiratory coupling, our analysis showed that nine out of twelve subjects presented obvious CRPS at SL, and phase synchronization emerged at 3000 m and 4000 m in all subjects. The change of CRPS was plotted in Figure 4. It illustrated the duration of phase synchronization increased with the altitude. *T* at 3000 m was not significantly different from that at SL. However, it increased significantly at 4000 m compared with the value at SL. The result indicated acute hypoxia had a significantly stronger effect on CRPS in normal subjects.

## Discussion

Acute exposure to hypoxia triggers autonomic mechanisms in cardiovascular and respiratory system. In this study, we not only investigated the changes of power spectrum and SampEn of heartbeat series, but also observed cardiorespiratory coupling through phase synchronization at each simulated altitude. The main finding in the present study is that acute hypoxia evokes vigorous CRPS, as evidenced by the longer values of *T*. Meanwhile, hypoxia can also lead to higher value of SampEn and decrease in power spectral HRV indices.

Acute hypoxia evokes several regulatory mechanisms of ANS. Spectral analysis of RRI series is considered as an effective tool to investigate autonomic activities. The effect of acute hypoxia on autonomic nervous activity is complicated and not fully understood. Previous studies that were revealed in different protocols showed that autonomic nervous activities were attenuated in hypoxic conditions, and that the sympathetic activities were predominant compared with the parasympathetic at high altitude [1,7,9,15]. In the present study, depression of HRV parameters in frequency domain might be the result of a general decline of ANS responses. The drastic increase of the LF/HF ratio in our results indicated the sympathovagal balance shifted toward sympathetic dominance through sympathetic activation and parasympathetic withdrawal in acute hypoxic exposure. The result implied the sympathetic HR control was relatively less blunted than the parasympathetic HR control. Therefore, the observed increase of resting HR in acute hypoxia was ascribed to the general attenuation of autonomic HR control and also the shift of autonomic balance.

Compared with the linear HRV analysis, nonlinear dynamics analysis is a powerful tool to understand biological characteristics, because nonlinear analyses of heart rate time series could not only complement the traditional time- and frequency-domain analyses but also provide essential information on human heartbeat dynamics. Richman and Moorman [30] introduced the sample entropy to quantify irregularity and complexity of analyzed sequences. Previous studies indicated that complexity of beat-to-beat variability was controlled by ANS [34]: parasympathetic blockade could reduce heartbeat complexity [35]; parasympathetic activation increased complexity [36]. On the other hand, sympathetic excitation by pharmacological [35] or physiological method [34] reduced complexity and sympathetic blockade with propranolol increased irregularity [37]. Heffernan et al. [38] found no changes in spectral HRV parameters after resistance training accompanied with significant increase in SampEn. However, Javorka et al. [39] found heart rate complexity was slightly reduced after exercise. These experiments indicated that parasympathetic and sympathetic tone modulated cardiovascular nonlinear activities in normal subjects. However, the exact contributions of sympathetic and parasympathetic branches to nonlinear fluctuations in heartbeat series required more studies to separate. On the other hand, Vigo et al. [7] demonstrated that changes in nonlinear HRV parameters might not be directly associated with the fluctuations of heartbeat, especially when the heart rate increased. Our result that the increase of SampEn in acute hypoxia was accompanied with parasympathetic depression and predominance of sympathetic tone supported the proposition of Porta et al. [34] that irregularity of heartbeat series reflected general sympathovagal balance. This indicated that acute hypoxia enhanced autonomic modulation of heartbeat irregularity, reflecting the increase in sympathetic activity and/or the decline in parasympathetic autonomic control.

Our results obtained from healthy subjects showed changes in the degree of CRPS at different simulated altitudes. The results demonstrated that autonomic regulation with different physiological stress strongly influenced cardiorespiratory coupling. In different simulated altitudes, we found that phase synchronization, which was a complicated nonlinear physiologic coupling, increased significantly in hypoxia. The observation that phase synchronization was present at different altitudes in our study provided an evidence for the existence of CRPS in healthy relaxed subjects. However, the total episodes of synchronization did not exceed 90 seconds within the 10 minutes recordings in all subjects at SL. This was shorter than Schafer et al. [19,40] results that within the 30 minutes segment the longest duration of synchronization was more than 4 minutes. This discrepancy may be ascribed to the different composition of subjects (athletes vs. non-athletes). Further, we analyzed phase synchronization in different simulated altitudes and demonstrated the total synchronization times at 3000 m was no significantly different from that at SL, but it increased significantly at 4000 m. The result indicated CRPS in hypoxia was more pronounced than that at SL. Hypoxia was associated with physiologic regulation characterized by different neuro-autonomic tone and levels of sympathovagal balance [27]. A higher degree of CRPS in acute hypoxia when sympathetic excitation accompanied with parasympathetic depression suggested that cardiorespiratory coupling was intensively affected by neuro-autonomic regulation. Further, this relation between CRPS and autonomic nervous activity agreed with our observation that the trends of the LF/HF ratio were consistent with *T* in hypoxia.

Phase synchronization between heartbeats and breathing was a manifestation of the temporal organization and regulation of cardiac and respiratory rhythms owning to their central coupling between cardiovascular and respiratory neural activities [32]. Because the variability and nonlinear properties of cardiac and respiratory system varied with physiological state and with environmental stimulus, quantifying CRPS in different simulated altitudes may provide insight into how physiologic adjustment affected cardiorespiratory coupling. Although HR increased significantly at 3000 m, neither *T* nor breathing rate changed significantly in our result. At 4000 m, both HR and respiratory rate augmented significantly than that at SL as well as phase synchronization time. These results supported the hypothesis of Rosenblum [20] that cardiopulmonary interaction was unidirectional from breathing to cardiovascular system. The explanation was suggested in the following. Because the cardiac influence on respiration was weak and frequency independent [20], the increase of HR was limited to influence phase synchronization. At the same time, for the low breathing frequencies (RespR < 0.5 Hz) the respiratory driving effect was relatively strong compared to the strength of the cardiac influence [20].

In our results, the vigorous CRPS in hypoxia was concomitant with the increase of heartbeat series SampEn and the shift of sympathovagal balance. This indicated more complex interconnections between the cardiac and respiratory system in hypoxic condition. There would be some different effects of autonomic regulation in terms of the modulation of cardiorespiratory coupling in acute hypoxia. The decrease of spectral HRV parameters in hypoxia could be explained as a general decline of the autonomic nervous activities. Reduction in HRV led to decreasing the responses of ANS and being unable to adapt to challenging external and internal stimuli [1]. On the other hand, cardiorespiratory system was inherently nonstationary and contained only quasiperiodic oscillations [26]. We observed that the coupling was more pronounced when the complexity of heartbeat series increased in acute hypoxia. This meant the features of cardiac dynamics were more irregularity and nonlinearity in hypoxia. Therefore, the more irregularity RRI series was, the higher the probability that heartbeats consistently occurred at the same respiratory phase for continuous breathing cycles was. Moreover, the cardiopulmonary system was a thermodynamic open system [41], and the external disturbances on it could be considered as noise. The pronounced CRPS was associated with the decline of HRV, indicating that the low activities of ANS in hypoxia restrained autonomic responses to noise and accentuated the intrinsic rhythm of cardiac and respiratory system. The other possibility was the mechanical coupling between cardiac and respiratory system. This interaction was generated by mechanical stretch of the sinus node [42] and not blunted by neural control.

Mostly notably, this is the first study, to our knowledge, to investigate CRPS in hypoxia. Our results provide compelling evidence that the variability and nonlinear feature of the cardiac and respiratory systems change with physiologic conditions. Exposure time and hypoxic degree are two major variables in hypoxia [9]. Investigation on CRPS in different exposure protocols helps us to understand how physiological stress affects CRPS in healthy subjects. However, some limitations of this study need to be taken into account. The number of subjects was limited and only male subjects took part in the experiment. Studies with larger sample size, both genders and a wide age range are necessary to elucidate the potential changes of CRPS in different physiologic states and conditions.

## Conclusion

This study observed that HRV spectral parameters decreased and the complexity of heartbeat series increased in acute hypoxia. Moreover, cardiorespiratory coupling was investigated through phase synchronization during transitions in different simulated altitudes. The results suggested that CRPS, which was more vigorous in hypoxia, was a manifestation of cardiac and respiratory regulation due to their underlying coupling. This study is the first step to understand how physiological stress influences CRPS in healthy subjects. A thorough understanding of cardiorespiratory coupling in different physiological states and conditions may provide valuable information about mechanisms of physiological regulation, which would be explored in the following studies.

## Competing interests

The authors declared no conflict of interest.

## Authors' contributions

DZ contributed to experimental conception and design, acquisition, analysis and interpretation of data and drafting the manuscript. JS participated to coordination and helped to mathematic calculation and interpretation of data. ZZ has participated in data analysis and interpretation, and helped draft and revise the manuscript. MY conceived of the study and participated in its design and coordination. All authors read and approved the final manuscript.
